# Social Citizenship Through Out-of-Home Participation Among Older Adults With and Without Dementia

**DOI:** 10.1177/07334648221112425

**Published:** 2022-06-30

**Authors:** Sophie N. Gaber, Liv Thalén, Camilla W. Malinowsky, Isabel Margot-Cattin, Kishore Seetharaman, Habib Chaudhury, Malcolm Cutchin, Sarah Wallcook, Anders Kottorp, Anna Brorsson, Samantha Biglieri, Louise Nygård

**Affiliations:** 1 Division of Occupational Therapy, Department of Neurobiology, Care Sciences and Society, 27106 Karolinska Institutet, Huddinge, Sweden; 2 Department of Health Care Sciences, Marie Cederschiöld University, Stockholm, Sweden; 3 Department of Occupational Therapy, School of Social Work and Health, Lausanne (HETSL), 111832 University of Applied Sciences and Arts of Western Switzerland (HES-SO) Delémont, Switzerland; 4 Department of Gerontology, 1763 Simon Fraser University, Burnaby, BC, Canada; 5 144477 Pacific Northwest University of Health Sciences, Yakima, WA, USA; 6 Faculty of Health and Society, 59606 Malmö University, Malmö, Sweden; 7 School of Urban and Regional Planning, 7984 Ryerson University, Toronto, ON, Canada

**Keywords:** dementia-friendly communities, international, neighborhood, neurodegenerative diseases, outdoor environment

## Abstract

There is limited empirical knowledge about how older adults living with dementia enact their social citizenship through out-of-home participation. This study aimed: (a) to investigate out-of-home participation among older adults with and without dementia in four countries and (b) to compare aspects of stability or change in out-of-home participation. Using a cross-sectional design, older adults with mild-to-moderate dementia and without dementia, aged 55 years and over, were interviewed using the Participation in ACTivities and Places OUTside the Home questionnaire in Canada (*n* = 58), Sweden (*n* = 69), Switzerland (*n* = 70), and the United Kingdom (*n* = 128). Data were analyzed using descriptive statistics and a two-way analysis of variance. After adjustment for age, diagnosis of dementia and country of residence had significant effects on total out-of-home participation (*p* < .01). The results contribute to policies and development of programs to facilitate social citizenship by targeting specific activities and places.

**What this paper adds**

• A cross-national perspective of out-of-home participation among older adults living with and without dementia.
• After adjustment for age, diagnosis of dementia and country of residence had significant effects on out-of-home participation among the participants.

**Applications of study findings**

• We propose a strengths-based view of older adults living with and without dementia as they enact their social citizenship through participation in activities and places in their communities.
• To develop more targeted programs, policies, and built environment interventions for older adults living with and without dementia, there is a need to focus on maintaining participation in specific activities and places in the community.

## Background and Objectives

Out-of-home participation is considered to promote and maintain health and well-being among older adults living with and without dementia (Douglas et al., 2017; Evans et al., 2019), and yet existing substantive knowledge in this area and methodological understanding are limited. Health and well-being benefits associated with out-of-home participation include social interaction (Livingston et al., 2020), mental health promotion (Mackenzie & Abdulrazaq, 2021), cognitive stimulation (Evans et al., 2019), physical activity, and mobility (Odzakovic et al., 2020). However, health and well-being are more than individual concerns; they are also socially constructed (Douglas et al., 2017).

Increasingly, research suggests that older adults living with and without dementia participate in activities and places outside their home not only for health, functional and mobility benefits, but also as a way to enact their social citizenship (Bartlett, 2021; Nedlund et al., 2019). In dementia research, social citizenship refers to “a relationship, practice or status, in which a person with dementia is entitled to experience freedom from discrimination, and to have opportunities to grow and participate in life to the fullest extent possible” (Bartlett & O'Connor, 2010, p. 37). Our conceptualization builds on the theory of social citizenship which recognizes that older adults living with and without dementia are active agents with competencies, histories, rights, and responsibilities, which link the person to a community and country of residence (Bartlett & O'Connor, 2010).

A theory of social citizenship provides a lens, which helps understand the ways in which older adults, including those living with dementia, enact their agency and citizenship though participation in “ordinary places” (Nedlund et al., 2019). Older adults can enact their citizenship outside the home through their ongoing and everyday participation in “ordinary places” such as the neighborhood, a grocery store, and a friend or family member’s place (Bartlett, 2021; Fransen-Jaïbi et al., 2021). Activities are embedded in these places, and thus, activities and places are integrally linked. For the purposes of this study, participation is viewed as linking the person, place, and activities (Gan et al., 2021).

The present study’s conceptualization of social citizenship is aligned with a strengths-based view of out-of-home participation that draws on the lived experience of older adults and frames places outside the home as zones of mastery, opportunity, and challenge (Ward et al., 2021). This approach acknowledges the agency of older adults living with or without dementia to enact their social citizenship by choosing to participate in some activities and places, and not others; thus, focusing on out-of-home activities and places that are abandoned, as well as those that are maintained.

Both aging and living with a diagnosis of dementia can affect older adults’ out-of-home participation (Hedman et al., 2017; Nygård & Kottorp, 2014). These age-related changes such as decline in functional, sensory, physical, and cognitive capabilities may impact participation, for instance, due to reduced range of movement, stamina or coordination, which may inhibit mobility (Brorsson et al., 2020; Kuspinar et al., 2020; Mick et al., 2018), or exacerbate wayfinding or navigation related challenges (Wiener & Pazzaglia, 2021) while outside the home.

The complexity of out-of-home participation suggests that individuals’ cognitive status and age need to be considered in relation to other factors potentially influencing out-of-home participation, such as the “living environment” (Kuspinar et al., 2020; Ward et al., 2021). For the purposes of this study’s focus on out-of-home participation, the living environment encompasses the different activities and places where older adults may enact their social citizenship outside of their homes, including the neighborhood and community at large. There are structural and contextual aspects that influence the living environment; thus, opportunities for out-of-home participation may vary among different countries (Townsend et al., 2021). Structural and contextual aspects include city planning, urban design and walkability (Biglieri, 2018; Houston et al., 2020), proximity to nature (Sturge et al., 2021), socio-economic status (d'Orsi et al., 2014; Gaber et al., 2020; Wallcook et al., 2021), access to public transport and transport services (Kizony et al., 2020; Mouratidis, 2018), access to health care services including diagnostic and support services for older adults living with dementia and other age-related or disabling conditions (Gan et al., 2021).

In addition to structural and contextual aspects of participation in the living environment, there are also social aspects to consider. Cultural norms and values concerning age and dementia can impact out-of-home participation (Bartlett & Brannelly, 2019; Blackman et al., 2003). For older adults living with dementia, there can be additional social stigma which may increase the risk of stress, shame, and embarrassment in public places, leading them to avoid out-of-home participation (Blackman et al., 2003; O'Connor et al., 2018; Renn et al., 2021); this consequently diminishes the health and well-being benefits of out-of-home participation. Dementia-friendly communities encompass places and initiatives that seek to address these considerations by empowering and supporting the equal rights and resources of older adults living with dementia and their care partners, through several strategies including increasing public awareness, supportive programs and services, responsive social practices and adaptation of the physical environment (Biglieri, 2018; Gan et al., 2021; Wiener & Pazzaglia, 2021). Thus, dementia-friendly communities may facilitate opportunities for social citizenship. Nonetheless, there is insufficient knowledge on comparative out-of-home participation as an indication of social citizenship in various countries, and whether there are significant similarities or differences between older adults living with or without dementia.

Based on our conceptualization and earlier research, in this study, we hypothesized that (a) older adults living with dementia would participate in fewer activities and places outside the home than older adults living without dementia; (b) older adults living with dementia would have abandoned a higher number of activities and places outside the home between the past and present than older adults living without dementia; and (c) that having a diagnosis of dementia and the country of residence would have a significant effect on out-of-home participation, when controlling for age. Thus, this study aimed (a) to investigate out-of-home participation among older adults living with and without dementia in four countries and (b) to compare aspects of stability or change in out-of-home participation across those countries.

## Methods

### Study Design and Setting

A cross-sectional design was used to investigate the study aims and hypotheses in four countries, and the authors adhered to the Strengthening the reporting of observational studies in epidemiology (STROBE) checklist for cross-sectional studies (von Elm et al., 2008). This study is part of a cross-national project exploring out-of-home participation among older adults living with and without dementia in different countries. Table 1 provides an overview of the contextual characteristics pertaining to the Canadian, Swedish, Swiss, and UK samples. The rationale for the selection of these countries is twofold. First, the four countries are relatively comparable being high income countries located in the northern hemisphere with universal health care systems and dementia strategies in place (Department of Health, 2009, 2012; Federal Office of Public Health [Office Fédéral de la Santé Publique], 2018; Public Health Agency of Canada, 2019; Swedish Ministry of Social Affairs [Socialdepartementet], 2018). The four countries share a similar proportion of the older adult population living with dementia (Alzheimer Europe, 2020; Public Health Agency of Canada, 2018), and each country has age-friendly (World Health Organization., 2015) and dementia-friendly policies in place. Despite these shared characteristics, there are differences among the four countries, such as different public policies and programs in care and service needs of older adults. Also, the built environmental characteristics of the living environments are varied, for example, lower residential density in Canada compared to Sweden. Given the variability, we hypothesize there would be differences in out-of-home participation in these countries. Second, there is a pragmatic rationale as the collaborative project includes investigators in all four countries with experience in conducting research with older adults living with dementia. Thus, the cross-national sample arose organically through the research networks and provided an opportunity to investigate whether social citizenship enacted through out-of-home participation varies between the different countries.

**Table 1.** Overview of the Contextual Characteristics of the Sample of Older Adults Living With and Without Dementia in the Four Participating Countries.

Variable	Category	Canada	Sweden	Switzerland	UK (England)
Data collection regions		Vancouver	Stockholm region	French speaking regions of western Switzerland	Cumbria, Greater Manchester, London (North East, South West London) region
Data collection environments	Urban/suburban	58 (100%)	30 (44.12%)	17 (24.29%)	98 (76.56%)
	Rural/semi-rural	0 (0%)	38 (55.88%)	53 (75.71%)	30 (23.44%)
Recruitment settings for older adults living with dementia	Older adults living with dementia	Memory clinics	Memory clinics and open, community-based settings	Memory clinics	National Health care Service (NHS) including memory clinics
	Older adults living without dementia	Senior centers	Community-based leisure and social groups and senior centers	Senior associations	Community-based leisure and social groups and senior centers
National public transport services		British Columbia transportation system offers public transportation concessions and a bus pass for older adults (65+)	Public transportation concessions for older adults (65+)	Reductions for seniors in some communes, free pass for older adults	Nationwide Free older person’s bus pass and regional public transportation concessions, that is, the London Freedom Pass for older adults and/or disabled persons
National dementia population		432,010 (1.19%)	168,243 (1.66%)	137,344 (1.62%)	1,031,396 (1.56%)
National dementia strategy		A Dementia Strategy for Canada: Together We Aspire (2019)	Swedish National Guidelines on Dementia (2017), and the Swedish National Dementia Strategy (2018)	Swiss National Dementia Strategy (2013), evaluation (2016), and Dementia Platform (2019)	National Dementia Strategy for England: Living well with dementia (2009), the revised implementation programme (2012), and the Prime Minister’s Challenge on Dementia (2015)

*Notes.* Missing data: Data collection environments (1 older adults living with dementia in the Swedish sample). National dementia population statistics (Sweden, Switzerland, UK) according to Alzheimer Europe (2020); National dementia population statistics (Canada) according to the Public Health Agency of Canada (2018).

### Participants

A total of 325 older adults living with and without dementia were included in this study (see Table 2 for sample details). The sample consisted of 162 older adults living with dementia recruited from Canada (*n* = 28), Sweden (*n* = 35), Switzerland (*n* = 35) and the United Kingdom (UK, *n* = 64), and a further 163 older adults living without dementia (i.e., no known cognitive impairment) from Canada (*n* = 30), Sweden (*n* = 34), Switzerland (*n* = 35) and the UK (*n* = 64). The sample size and power were calculated using an earlier study with the Swiss sample, indicating the need for a sample size of approximately 19 participants for each sub-sample (α = .05; power = .8).

**Table 2.** Background Characteristics of the Sample (*n* = 325).

Variable	Category	Sub-sample of older adults living with dementia (*n* = 162)				Sub-sample of older adults living without dementia (*n* = 163)				Test statistic	
		Count	%	Median	IQR	Count	%	Median	IQR	*χ*^*2*^ *or U*	*P*
Sex	Male	79	48.77			65	39.88			2.6	NS^a^
	Female	83	51.23			98	60.12				
Age group	≤64 years	11	6.96			28	17.18			10.63	NS^a^
	65–74 years	50	31.65			59	36.2				
	75–84 years	71	44.94			56	34.36				
	≥85 years	26	16.46			20	12.27				
Level of education	Primary and/or secondary school	35	21.88			22	13.5			6.96	NS^a^
	High school/GED/Apprenticeship	84	52.5			80	49.08				
	Degree from university/college	41	25.63			61	37.42				
Living conditions	Alone	60	37.01			83	50.92			6.36	NS^a^
	Co-habiting	102	62.96			80	49.08				
Supporting person available	No	9	5.66			44	27.16			26.91	<.001^a^
	Yes	150	94.34			118	72.84				
Driving car yourself	No	120	74.07			62	38.04			42.82	<.001^a^
	Yes	42	25.93			101	61.96				
Transportation service	No	120	74.53			152	93.25			21.06	<.001^a^
	Yes	41	25.47			11	6.75				
MoCA	Score (0–30)			20	16–22			26	24.50–28	1224.5	<.001^b^
MMSE	Score (0–30)			21	19–22.75			29	28–30	.0	<.001^b^

*Notes.* GED: General Educational Development exam; IQR: Interquartile range; MMSE: Mini-Mental State Examination (for the Canadian sample); MoCA: Montreal Cognitive Assessment (for the Swedish, Swiss, and UK samples); NS: non-statistically significant difference.

^a^Analyzed with Pearson’s χ^2^.

^b^Analyzed with the Mann–Whitney *U* test. Missing data: Age (four older adults living with dementia), level of education (two older adults living with dementia), supporting person available (three older adults living with dementia and 1 older adult living without dementia), transportation service (one older adults living with dementia), MMSE (16 older adults living with dementia and six older adults living without dementia).

The same inclusion and exclusion criteria were adopted across the four countries. Older adults were included if they were aged 55 years and above, living at home in their community, and they could communicate for themselves during an interview. For those living with dementia, a diagnosis of dementia was given by a physician and the participants were recruited from memory clinics (Canada, Sweden, snd Switzerland) and the National Health Service (UK). The data collectors used the brief cognitive screening tools that were commonly used in clinical practice at each recruitment site. Thus, the data collectors in Sweden, Switzerland, and the UK used the Montreal Cognitive Assessment (MoCA), whereas the data collectors in Canada used the Mini-Mental State Examination (MMSE). The older adults living without dementia were recruited through senior centers (Canada), senior associations (Switzerland), and local networks, such as community-based leisure and social groups (Sweden, UK). All participants provided their verbal and written informed consent for inclusion before they participated. This research was granted ethical approval by the Office of Research Ethics at Simon Fraser University (2017s0052) for the Canadian sample, the Regional Board of Research Ethics (2015/77-31-5) for the Swedish sample, the Commission cantonale d’éthique de la recherche sur l'être humain in Lausanne (protocol 452/15) for the Swiss sample, and the Health Research Authority (IRAS project ID: 215654, REC reference: 17/SW/0091) for the UK sample. This study adhered to the principles of the Helsinki Declaration (World Medical Association, 2013).

### Measures and Materials

#### Total Out-of-Home Participation

This paper used the Participation in ACTivities and Places OUTside Home Questionnaire (ACT-OUT) as a novel tool to explore social citizenship through the older adults’ patterns of out-of-home participation in activities and places (Margot-Cattin et al., 2019). The ACT-OUT questionnaire was developed with older adults living with and without dementia and an in-depth explanation of the development process in three languages (English, French, and Swedish) can be found elsewhere (Margot-Cattin et al., 2019). Earlier studies using the ACT-OUT questionnaire in different countries (Chaudhury et al., 2021) and in conjunction with other instruments (Gaber et al., 2021; Margot-Cattin et al., 2021; Wallcook et al., 2021) contribute to the validity of the ACT-OUT questionnaire. Further psychometric testing is underway.

The ACT-OUT questionnaire has three parts. Part one maps past and present participation in 24 types of places and activities, part two elicits more detail about the activities performed in two places for each domain, and part three enquires about perceived risks when participating outside the home. For the purposes of this study, only part one was utilized to map participation according to four domains: (A) Consumer, administrative and self-care places (*n* = 6), (B) Places for medical care (*n* = 5), (C) Social, cultural and spiritual places (*n* = 6) and (D) Places for recreational and physical activities (*n* = 7). The participants responded yes (1) or no (0) to participating in each type of place in the past and present and these responses were used to calculate total out-of-home participation scores out of a maximum of 24 places. The past and present time-points for participation were self-determined by each participant.

#### Factors

Sociodemographic and other background characteristics collected using a demographic questionnaire were selected as factors (i.e., categorical variables) (Table 2). These factors were selected due to the statistically significant differences between the sub-samples of older adults living with and without dementia (i.e., supporting person available, driving a car yourself, and access to a transportation service) and based on earlier research which indicates that age (Hedman et al., 2017), cognitive status or diagnosis of dementia (Nygård & Kottorp, 2014) and differences between countries (Gan et al., 2021) may influence out-of-home participation.

### Procedures

#### Data Collection

Data were collected between 2015 and 2017. For the ACT-OUT and demographic questionnaires, the investigators elicited questions and recorded the participants’ responses using the data collection tools. Data were collected using face-to-face interviews at the participants’ homes or another location of their choice. Participants could elect to have a significant other (i.e., spouse, partner, or any caregiver such as a sibling, or adult child) present for support during the interview, but not for proxy-reporting. To ensure a flexible approach to meet the participants’ needs, abilities and routines, the interview procedure could be spread across three different sessions with each session lasting under 90 minutes.

#### Data Analysis

Analyses first compared stability and change in out-of-home participation between the sub-samples of older adults living with and without dementia in the four countries using descriptive statistics (hypotheses a and b). The next stage of analyses investigated the effects of the factors using univariate analyses and a multivariate model investigating whether having a diagnosis of dementia and the country of residence would have a significant effect on out-of-home participation, when controlling for age (hypothesis c).

Differences between the sub-samples of older adults living with and without dementia were investigated using the independent samples t-test. Differences comparing past and present participation within each sub-sample were tested using the dependent samples t-test. To minimize the risk of Type I errors, the significance value was set at *p* < .01 for all analyses, including Bonferroni corrections. The results were interpreted according to the effect size thresholds for partial eta squared (.01 = small; .06 = medium; .14 = large effect) (Cohen, 1988).

Preliminary tests revealed no violation of the assumptions to ensure the data fulfilled criteria for a two-way analysis of variance (ANOVA) i.e., normality, linearity, homogeneity of variances or collinearity. One outlier was identified; however, sensitivity analyses showed no significant effect on the results when the outlier was removed from the models; thus, it was included in the analyses. Univariate analyses were performed, followed by a backward selection procedure to identify, and to remove, statistically redundant variables with a significance value less than .01 (supporting person available, driving a car yourself, transportation service). The backward selection procedure was motivated by the lack of earlier research regarding the relationship between the variables. In the final multivariate model, we conducted a two-way ANOVA to investigate the main and interaction effects of diagnosis group and country of residence on total out-of-home participation, when controlling for age group (Table 5). Moreover, we performed pairwise comparisons using the Least Significant Difference test, to determine statistically significant differences in total out-of-home participation between diagnosis, country of residence and age groups (Table 6)

## Results

We begin by presenting the background characteristics of the two sub-samples. Next, we present the descriptive results in relation to hypotheses a and b, followed by the main results from the statistical model, to address hypothesis c.

### Background Characteristics of the Sub-Samples

Table 2 presents comparisons of background characteristics and identifies the significant differences between the sub-samples of older adults living with and without dementia.

### Total Out-of-Home Participation (Hypothesis a)

Total out-of-home participation was significantly lower for the sub-sample of older adults living with dementia compared to the sub-sample of older adults living without dementia. Table 3 reveals significantly lower participation among the sub-sample of older people living with dementia according to Domains A (Consumer, administration, and self-care places), C (Social, spiritual, and cultural places), and D (Places for recreation and physical activities). However, the difference was non-significant between the sub-samples for Domain B (Places for medical care).

**Table 3.** Mean Out-of-Home Present Participation in the ACT-OUT Questionnaire According to the Total Number of Places and for Each Domain A-D.

Variable	Values	Sub-sample of older adults living with dementia (*n* = 162)	Sub-sample of older adults living without dementia (*n* = 163)	*t*	*df*	*P*	Cohen’s *d*	99% CI
Total out-of-home participation (*n* = 24)	Mean (min-max)	15.19 (2–22)	17.6 (8–23)	6.82	299.89	<.001^a^	.76	.46, 1.05
	SD	3.58	2.71					
Domain a (*n* = 6)	Mean (min-max)	4.43 (0–6)	5.24 (1–6)	5.79	263.48	<.001^a^	.64	.35, .94
	SD	1.52	.92					
Domain B (*n* = 5)	Mean (min-max)	2.84 (0–5)	2.97 (0–5)	1.3	315.07	NS^a^	.15	−.14, .43
	SD	.97	.83					
Domain C (*n* = 6)	Mean (min-max)	3.81 (0–6)	4.57 (1–6)	5.09	323	<.001^a^	.57	.27, .86
	SD	1.42	1.25					
Domain D (*n* = 7)	Mean (min-max)	4.1 (0–7)	4.82 (1–7)	4.35	323	<.001^a^	.48	.19, .77
	SD	1.54	1.4					

*Notes.* CI: confidence intervals; *df*: degrees of freedom; NS: non-statistically significant difference; SD: standard deviation.

Domain A: Consumer, administration, and self-care places.

Domain B: Places for medical care.

Domain C: Social, spiritual and cultural places.

Domain D: Places for recreation and physical activities.

^a^analyzed with the independent t-test.

### Stability and Change in Out-of-Home Participation According to Place Type (Hypothesis b)

For both sub-samples, present participation generally decreased from past participation across the 24 place types. Table 4 shows a statistically significant decrease from past to present participation for 21 out of 24 place types among the sub-sample of older adults living with dementia. Similarly, but to a lesser degree, 14 out of 24 place types were associated with a statistically significant decrease in present participation from past participation among the sub-sample of older adults living without dementia. When comparing past participation between the two sub-samples a significant difference was identified in only one place type, Day care. The older adults living with dementia reported significantly higher past participation in Day care compared to the sub-sample of older adults living without dementia. This relative stability in terms of declining participation differed from present participation, where only 11 out of 24 place types showed significant differences between the sub-samples. Across all domains, present participation was significantly higher in one place type (Day care) and significantly lower in 10 place types (Mall, supermarket; Small store; Pharmacy; Bank, post office; Doctor’s office; Dentist’s office; Senior center, social club; Entertainment, cultural place; Forest, mountain, lake, sea; and Sports facility) among the sub-sample of older adults living with dementia compared to the sub-sample without dementia. Present participation in the Neighborhood remained relatively high for both older adults living with and without dementia and there was no statistically significant difference between the sub-samples.

**Table 4.** Percent Difference in Past and Present Out-of-Home Participation in the ACT-OUT Questionnaire According to Each Type of Place.

Place type	Sub-sample of older adults living with dementia		Sub-sample of older adults living without dementia		Difference in past/present participation between sub-samples^b^
	Past/present participation	*p*^a^	Past/present participation	*p*^a^	
Domain A: Consumer, administration, and self-care places (*n* = 6)					
Small grocery store	86/72	<.001	89/80	<.001	NS/NS
Mall, supermarket	98/83	<.001	98/98	NS	NS/<.001
Small store	92/75	<.001	91/88	NS	NS/.002
Pharmacy	98/77	<.001	95/89	NS	NS/.004
Hairdresser	90/79	<.001	86/83	NS	NS/NS
Bank, post office	96/61	<.001	97/88	<.001	NS/<.001
Domain B: Places for medical care (*n* = 5)					
Doctor’s office	85/80	NS	92/91	NS	NS/.007
Hospital, health center	84/62	<.001	78/71	.004	NS/NS
Dentist’s office	95/78	<.001	94/90	NS	NS/.006
Therapy	62/35	<.001	71/42	<.001	NS/NS
Day care	21/29	NS	6/4	NS	<.001/<.001
Domain C: Social, spiritual, and cultural places (*n* = 6)					
Friend, family member’s place	97/87	<.001	96/91	NS	NS/NS
Restaurant, café, bar	94/88	NS	94/90	NS	NS/NS
Senior center, social club	72/41	<.001	82/73	<.001	NS/<.001
Building for worship	72/52	<.001	79/61	<.001	NS/NS
Cemetery, memorial place	71/54	<.001	68/56	<.001	NS/NS
Entertainment, cultural place	93/62	<.001	93/87	NS	NS/<.001
Domain D: Places for recreation and physical activities (*n* = 7)					
Garden	86/75	<.001	87/74	<.001	NS/NS
Park, green area	85/68	<.001	86/71	<.001	NS/NS
Forest, mountain, lake, sea	93/59	<.001	97/77	<.001	NS/<.001
Cottage, summer house	72/34	<.001	68/44	<.001	NS/NS
Neighborhood	96/89	<.001	93/88	.008	NS/NS
Sports facility	74/25	<.001	75/48	<.001	NS/<.001
Transportation center	93/66	<.001	96/79	<.001	NS/NS

*Notes.* NS: non-statistically significant difference.

^a^Dependent samples t-test.

^b^independent samples t-test.

### Results from the two-way analysis of variance (ANOVA) (Hypothesis c)

When controlling for age group, the interaction effect on total out-of-home participation between diagnosis group and country of residence was statistically significant, *F* (3, 310) = 4.46, *p* = .004, although the effect size was small (partial eta squared = .04). Furthermore, there was a statistically significant main effect on total out-of-home participation for diagnosis group, *F* (1, 310) = 53.63, *p* < .001, with a large effect size (partial eta squared = .15) and for country of residence, *F* (3, 310) = 5.79, *p* < .001, with a small effect size (partial eta squared = .05) (Table 5).

**Table 5.** Results From ANOVA.

	Outcome: Total present out-of-home participation (univariate models)				Outcome: Total present out-of-home participation (final multivariate model)			
Variable	*Df*	SS	*F*	Partial η^2^	*df*	SS	*F*	Partial η^2^
Diagnosis group	1	469.45	46.54**	.126	1	491	53.63**	.147
Country of residence	3	137.36	4.09*	.037	3	159.01	5.79**	.053
Age group	3	177.36	5.34*	.048	3	157.39	5.73**	.053
Supporting person available	1	53.58	4.67	.014				
Driving a car yourself	1	221.69	20.42**	.059				
Transportation service	1	68.55	6.03	.018				
Interaction effect								
Diagnosis group x country of residence					3	122.41	4.46*	.041
Error					310	2838.08		
Total					321	90,369		

*Notes.* A two-way analysis of variance (ANOVA) was performed; *df* = degree of freedom; Partial η^2^ = partial eta squared; SS = sum of squares.

*p < .01. **p < .001.

### Pairwise Comparisons Between Country of Residence and Age Groups

Pairwise comparisons indicated that the mean total out-of-home participation was significantly higher for participants living without dementia than those living with dementia (*p* < .001). Mean total out-of-home participation was higher among participants living in Sweden compared to those living in Canada (*p* < .001) and the UK (*p* < .01) (Table 6). Pairwise comparisons between age group revealed significantly higher mean total out-of-home participation among participants aged 65–74 years compared with participants in the oldest age group (≥85, *p* < .001).

**Table 6.** Pairwise Comparisons of Total Out-of-Home Participation Between Diagnosis, Country of Residence, and Age Groups.

Variable	Comparison	Mean difference	99% CI
Diagnosis group			
	PwDG v. CG	−2.62**	−3.56, −1.70
Country of residence			
Canada	Canada versus Sweden	−2.03**	−3.47, −.59
	Canada versus Switzerland	−1.17	−2.63, .29
	Canada versus UK	−.46	−1.75, .83
Sweden	Sweden versus Switzerland	.86	−.48, 2.2
	Sweden versus UK	1.57*	.40, 2.75
Switzerland	Switzerland versus UK	.71	−.47, 1.89
UK			
Age group			
≤64	≤64 versus 65–74	−.47	−1.97, 1.03
	≤64 versus 75–84	.43	−1.07, 1.93
	≤64 versus ≥ 85	1.75	−.04, 3.54
65–74	65–74 versus 75–84	.9	−.14, 1.94
	65–74 versus ≥ 85	2.22**	.81, 3.63
75–84	75–84 versus ≥ 85	1.33	−.04, 2.69
≥85			

*Note.* CI = confidence intervals; PwDG = participants living with dementia group; CG = comparison group.

*p* < .01. **p < .001.

## Discussion and Implications

The older adult study participants living with and without dementia participated in activities and places outside the home which can be seen as a way of enacting their social citizenship. However, the data from this study support our hypothesis (a) that older adults living with dementia participated in fewer activities and places outside the home than the older adults living without dementia.

The close examination of the older adults’ participation in domains and places of the ACT-OUT questionnaire in this study contributes increased understanding of older adults’ social citizenship by being engaged in the community. The pattern of significantly lower out-of-home participation among older adults living with dementia compared to those living without dementia was evident in the following place domains: Consumer, administration, and self-care places; Social, spiritual and cultural places; and Places for recreation and physical activities. However, there was no statistically significant difference in participation in Places for medical care between the sub-samples. Participation in Places for medical care is important for older adults, particularly those living with a diagnosis of dementia (Bayat et al., 2021). However, there may be detrimental consequences for the older adults’ social citizenship, health, and well-being, when they are no longer able to participate in those activities at the places that have been abandoned over time (Douglas et al., 2017; Evans et al., 2019). Further research is required to understand the individual level cognitive, perceptual and affective challenges, and socio-environmental barriers in the community, that may influence the lower out-of-home participation in the various types of activities and places, among older adults living with dementia. We also need to have a more in-depth and nuanced understanding of the relevance and meaning of lowered participation in maintaining or enacting social citizenship.

Regarding aspects of stability and change in out-of-home participation, the results support our hypothesis (b) that the older adults living with dementia abandoned a higher number of activities and places outside the home between the past and present than the older adults without dementia. Despite the decreases in out-of-home participation, both older adults living with and without dementia continued to participate in a variety of places. For older adults living with dementia, their experiences of socially rich “third places” in the community decline more over time than other older adults, which can exacerbate negative health effects due to less social stimulation (Oldenburg, 1989). “Third places” are places that provide opportunities for social engagement outside of home (first place) or work (second place) (Oldenburg, 1989). Participation among older adults living with dementia may decline in “third places,” including Social, spiritual and cultural places such as going to a Restaurant, café or bar, and visiting a Senior center or social club, or Places for recreation and physical activities such as sitting in a Park, green area. These represent a range of places for different purposes, levels of social engagements and activities that can provide an opportunity to be social, to observe and to talk to other people without the burden of formal social interactions which older adults living with dementia may perceive as challenging (Burton & Mitchell, 2006). Thus, future research may benefit from a deeper exploration of participation in these “third places” among older adults living with dementia (Mouratidis, 2018).

The finding that older adults living with dementia retained some place types and abandoned others underscores the need to conduct in-depth research on the socio-environmental characteristics and processes of engagement in specific place types. Table 4 shows that present participation was significantly lower in 10 place types among the sub-sample of older adults living with dementia compared to the sub-sample without dementia. The significantly lower participation in these 10 place types may be due to various reasons, including the preferences of the older adults themselves. However, it may also be due to accessibility issues or insufficient opportunities to participate in activities in these place types (Gan et al., 2021; Houston et al., 2020). Earlier research focused on participation in grocery stores and supermarkets suggests that older adults living with dementia experience increased challenges related to both their personal capabilities and the characteristics of the place which can limit participation (Brorsson et al., 2020).

The 10 place types at greater risk of being lost among older adults living with dementia point to the importance for developing responsive policy, services and programs for social practices, built environmental features, and organizational commitment in these place types. Increased knowledge is needed about the nature of experience of those activities and places where there was lower participation among the older adults living with dementia, to explore the meaning of lower out-of-home participation in relation to social citizenship and to promote cohesion between person and place through age-friendly communities in general and dementia-friendly communities specifically (Gan et al., 2021). To facilitate social citizenship, communities should consider the activities and places that older adults living with dementia value or need to participate in their everyday lives, but also whether older adults living with dementia can be supported to participate in these places (Houston et al., 2020; Sturge et al., 2021). Thus, this study contributes to the knowledge base regarding the types of activities and places which may benefit from targeted interventions and adaptations to enable older adults to enact their social citizenship through out-of-home participation.

We can also accept our hypothesis (c) that having a diagnosis of dementia and the country of residence have a significant effect on out-of-home participation, when controlling for age among our sample. The statistically significant interaction effect suggests that the experience of living with dementia may vary between countries and reinforces the call for research to focus on other aspects of participation, such as contextual consideration, in relation to the older adults’ diagnosis of dementia (Chaudhury & Oswald, 2019; Gan et al., 2021). More specifically, pairwise comparisons indicated that the mean total out-of-home participation was significantly higher for the older adults living with and without dementia in Sweden compared with those living in Canada and the UK. Further research is needed to unpack these cross-national aspects of out-of-home participation on a more granular level, including differences in experiences of stigma, whilst controlling for differences between samples.

The mean total out-of-home participation was significantly higher among participants aged 65–74 years than participants in the oldest age group (i.e., ≥85 years), and this corroborates earlier research linking increased age with decreased participation in activities among older adults (Hedman et al., 2017; Henning et al., 2021). However, it is somewhat surprising that the highest mean total out-of-home participation was among participants aged 65–74 years as opposed to the youngest age group (i.e., ≤64 years). This age group corresponds to the age of retirement among the four countries, and thus, retirement-related changes in out-of-home participation should be explored in future research.

### Limitations

The interpretation and contribution of this study’s results should be considered according to the following limitations. Due to the cross-sectional study design, it was possible to identify associations but not causal relationships among the study factors. Each participant interpreted past participation individually and retrospectively. Thus, the heterogeneity of responses is a limiting factor for comparisons across individual participants. The relatively small sample size recruited using purposive sampling was sufficient to meet the assumptions of the statistical models; however, it may limit the generalizability of the results particularly due to contextual differences between countries (Table 1). The results from this exploratory study may be investigated further employing a larger sample size, with balanced groups, and randomized sampling methods.

## Conclusion

This study has proposed a strengths-based view of the older adults living with and without dementia who enacted their social citizenship through participation in activities and places in their communities. The results underline the importance to consider not only older adults’ diagnosis of dementia, but also structural and contextual aspects of their country of residence which may also significantly impact out-of-home participation. More specifically, to enable the social citizenship of older adults, it is important to closely examine the types of activities and places where older adults living with dementia had significantly lower participation than older adults living without dementia, and to identify implications for responsive programs, policies and built environmental interventions.

## ORCID iDs

Sophie N. Gaber https://orcid.org/0000-0001-5804-0433

Liv Thalén’s https://orcid.org/0000-0002-1195-7946

Camilla Malinowsky’s https://orcid.org/0000-0002-6938-5282

Isabel Margot-Cattin’s https://orcid.org/0000-0002-1999-1452

Kishore Seetharaman https://orcid.org/0000-0002-9323-5620

Habib Chaudhury https://orcid.org/0000-0001-5770-3776

Malcolm Cutchin https://orcid.org/0000-0002-5407-465X

Sarah Wallcook https://orcid.org/0000-0002-8861-7552

Anders Kottorp https://orcid.org/0000-0002-8976-2612

Anna Brorsson https://orcid.org/0000-0003-1834-3418

Samantha Biglieri https://orcid.org/0000-0001-6106-5738

Louise Nygård https://orcid.org/0000-0003-1813-7390
